# Pulsed Electric Field Treatment Modulates Gene Expression and Stress Responses in Fusarium-Infected Malting Barley

**DOI:** 10.3390/plants14050668

**Published:** 2025-02-21

**Authors:** Pavel Svoboda, Jaroslava Ovesná, Štěpán Helmer, Milena Stránská

**Affiliations:** 1 Department of Molecular Genetics, Czech Agrifood Research Center (former Crop Research Institute), Drnovská 507, 161 06 Prague, Czech Republic; stepan.helmer@carc.cz; 2 Department of Plant Protection, Faculty of Agrobiology, Czech University of Life Sciences Prague, Kamycka 129, 165 00 Prague, Czech Republic; 3 Department of Food Analysis and Nutrition, Faculty of Food and Biochemical Technology, University of Chemistry and Technology, Technicka 3, 166 28 Prague, Czech Republic; milena.stranska@vscht.cz

**Keywords:** *Hordeum vulgare*, malting, pulsed electric field, *Fusarium* infection, transcriptomics, stress response, gene expression

## Abstract

Malting is a critical step in barley (*Hordeum vulgare*) processing, transforming grain into a key raw material for brewing and food production. However, the process is often compromised by *Fusarium* spp., pathogens responsible for Fusarium Head Blight, which reduces grain quality and safety. Pulsed electric field (PEF) treatment, a promising non-thermal technology, has been studied for its potential to inactivate microbial pathogens and mitigate infection-related stress. In this study, we investigated transcriptional responses in barley infected with *Fusarium* spp. during malting, both with and without PEF treatment. RNA sequencing identified over 12,000 differentially expressed genes (DEGs) across four malting stages, with the third stage (24 h of germination) showing the highest transcriptional activity. DEGs were significantly enriched in pathways related to oxidative stress management and abscisic acid signaling, underscoring their importance in stress adaptation. Barley treated with PEF exhibited fewer DEGs in later malting stages compared to untreated samples, suggesting that PEF alleviates stress induced by both *Fusarium* infection and the malting process. Enrichment analysis further revealed that PEF treatment up-regulated stress-related pathways while down-regulating genes associated with photosynthesis and cell wall biogenesis. These findings provide novel insights into barley stress responses during malting and highlight the potential of PEF as a tool for enhancing malt quality under stress conditions.

## 1. Introduction

Barley is the fourth most cultivated crop globally [1] and serves as the primary raw material for malting and brewing processes [2]. Various factors such as genotype, growing conditions, and soil quality significantly influence the quality and performance of barley malt during brewing [3]. 

Barley germination is a dynamic process involving a series of signaling pathways and biochemical changes that transform a metabolically inactive dry seed into an actively growing plant. Germination begins with water imbibition, which triggers the resumption of metabolism, particularly the synthesis and degradation of RNA and proteins [4]. Gibberellins (GAs) and abscisic acid (ABA) play key roles in regulating this process. ABA plays a crucial role in maintaining seed dormancy and inhibiting germination, whereas GAs promote germination by inducing the synthesis of hydrolytic enzymes, such as α-amylase, which catalyze the breakdown of starch into metabolizable sugars [4]. Early in germination, signaling pathways are triggered and genes regulating transcription are activated, allowing enzyme synthesis and preparation for mobilization of storage compounds [5]. Peroxidases are expressed in the early phase of germination and are used to detoxify reactive oxygen species produced during metabolic recovery. They also promote cell wall remodeling by oxidation of phenolic compounds, allowing cell expansion and radical penetration through the endosperm. Their activity gradually decreases in the later stages of germination [6].

This process is accompanied by a loss of tolerance to desiccation and cell expansion due to changes in the cell wall. The post-germination phase is characterized by the activation of photosynthetic pathways and the transition to autotrophic nutrition [7]. These changes are accompanied by extensive transcriptional modifications and remodeling of the chromatin structure, which allow the expression of genes required for growth [4]. The entire germination process is the result of the coordinated action of phytohormones, enzymes, and regulatory pathways that ensure the efficient conversion of dry seed into young plants [7].

*Fusarium* fungi pose a significant threat to barley, impacting both crop yield and quality. One of the most damaging fungal diseases of cereals is Fusarium Head Blight (FHB) caused by *Fusarium culmorum*, *F. graminearum*, *F. poae*, *F. sporotrichioides*, etc. *Fusarium* species, such as *Fusarium graminearum* and *Fusarium culmorum*, are known for producing mycotoxins like deoxynivalenol, which can contaminate malting barley grains [8]. This contamination not only leads to economic losses for farmers but also raises serious health concerns as mycotoxins can persist through the malting and brewing processes [9]. *Fusarium*-infected barley can result in reduced germination rates and compromised enzymatic activity during malting, negatively affecting the overall quality of malt and subsequently the final beer product [10]. Moreover, mycotoxin-contaminated malt can introduce undesirable flavors and odors into the beer, impacting its marketability [9].

To combat this issue, mitigation strategies categorized into chemical, biological, and physical methods are employed. Chemical treatments, like fungicide applications, are effective against fungal contamination but pose challenges such as residue accumulation, fungal resistance, and environmental concerns [11]. Additionally, consumers increasingly demand safer, residue-free food production methods [12]. Biological control, using beneficial microorganisms such as *Pythium oligandrum*, offers a sustainable alternative by inhibiting fungal growth through competition and antimicrobial production [13,14]. However, environmental variability can limit its effectiveness compared to chemical approaches [13].

Physical methods are gaining interest as chemical-free solutions for fungal control. Techniques such as heat treatments [15], UV irradiation [16], cold plasma [17], and high-pressure processing [18] effectively reduce fungal contamination but can affect food quality. For instance, heat treatments may alter texture and nutritional properties, while UV irradiation has limited penetration [19].

Pulsed Electric Field technology has emerged as a promising non-thermal method for fungal control. By applying short bursts of high voltage, PEF induces electroporation in microbial cell membranes, significantly reducing fungal viability while preserving sensory and nutritional quality [20,21]. PEF is energy-efficient, scalable for industrial applications, and synergizes well with techniques like mild heating or refrigeration for enhanced antimicrobial effects [22,23,24]. Recent studies demonstrate PEF’s potential in reducing mycotoxin levels in cereals and improving food processing efficiency, such as heat and mass transfer [25,26,27].

To evaluate PEF’s effects, researchers use physical, biochemical, and molecular approaches. Physical assessments measure fungal load before and after treatment to confirm microbial inactivation [28,29]. Biochemical analyses ensure that food quality traits, including enzyme activity, antioxidant capacity, and nutritional value, remain intact [30,31]. Molecular tools, such as proteomics and transcriptomics, provide insights into gene and protein level regulation, identifying stress responses and metabolic pathways affected by PEF [23,24,32].

Here, we present a pilot large-scale study exploring the influence of PEF on the transcriptomic profile of *Fusarium*-infected barley sampled at several stages of the malting process. This study aims to investigate whether and how pulsed electric field (PEF) treatment modulates barley’s transcriptional response to *Fusarium* infection during malting. By analyzing gene expression dynamics across malting stages, we explore whether PEF induces stress adaptation mechanisms that alter the plant’s response to both biotic (*Fusarium*) and abiotic (malting-associated) stressors.

## 2. Materials and Methods

### 2.1. Plant Material and Sampling

Barley (*Hordeum vulgare* L.) cultivar Bojos, which is among the varieties recommended for the production of beer with the protected geographical indication (PGI) ‘České pivo’ due to its malting suitability and ability to meet specific quality standards [33], was used as the input material for the pulsed electric field (PEF) treatment and subsequent malting. Seeds were obtained from the breeding company Limagrain Central Europe Cereals, Ltd. The barley was grown on a farm field (plot area 40 m^2^) of the Agrifood Research Center (former Crop Research Institute) in Prague for the purpose of producing infected seed. Sowing rates, based on breeders’ recommendations, were 400 grains per m^2^ and inter-row spacing was 12.5 cm. Cultivar Bojos was inoculated with a mixture of *Fusarium* species, including a highly pathogenic strain of *Fusarium culmorum* (no. VURV-F 425), three strains of *F. graminearum* (no. VURV-F 354, VURV-F 357, VURV-F 361), two strains of *F. poae* (no. VURV-F 995, VURV-F 996), and three strains of *F. sporotrichioides* (no. VURV-F 146, VURV-F 205, VURV-F 239). All strains were obtained from the culture collection of microorganisms at the Czech Agrifood Research Center (officially recognized by the World Federation for Culture Collections). Before field inoculation, the *Fusarium* isolates were cultivated on sterile wheat under controlled conditions. After two weeks, the sporulating mycelia were dried. A spore suspension was prepared from sterile distilled water and the dried inoculum, following the method of Chrpova et al. [34]. This spore mixture was sprayed directly onto the experimental field plot, first at full flowering and again one week later. Mist irrigation was applied to promote fungal infection. After grain harvest, the presence of individual *Fusarium* species was quantified, as described in Section 2.4. For the subsequent malting experiments, two batches (one kilogram each) from harvested grains were prepared—one subjected to PEF treatment and the other left untreated as a control—and both were processed as described in Section 2.3.

### 2.2. Sample Treatment

Prior to malting, a portion of the seeds underwent pulsion electric field treatment, while another portion of seeds was left untreated as a control group. For the experiments, a PEF system with a cylindrical batch treatment chamber was used. The experimental conditions were as described by Prusova et al. [35]. Description follows:

One kilogram of barley was soaked for 10 min in a 0.05 M phosphate buffer. After pre-soaking, the barley was treated with PEF under the following conditions: voltage of 3.8 kV/cm, current of 200 A, 100 bipolar pulses, and a pulse width of 20 µs. The total specific energy delivered to the barley was 152 J/g. A control sample of one kilogram of barley was also soaked in the 0.05 M phosphate buffer for the same duration and malted under the same conditions.

(1) $$Q=\frac{U\times I\times t\times n}{m}$$

The specific energy delivered in J/g for each PEF treatment was calculated using Formula (1), where ‘*Q*’ represents the specific energy, ‘*U*’ is the voltage displayed on the screen, ‘*I*’ is the current, ‘*t*’ stands for the duration of one pulse, ‘*n*’ is the number of pulses multiplied by two, and ‘*m*’ is the total mass of the batch chamber contents. For each treatment, 20 g of barley were suspended in the electrolyte, and the process was repeated 50 times.

### 2.3. Malting Conditions

**Figure 1:**
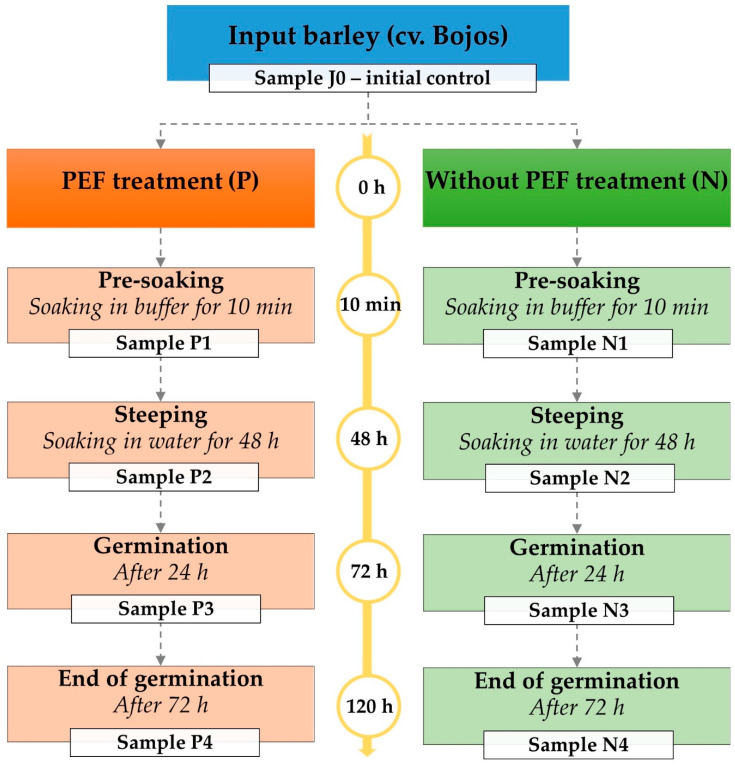
Schema of the experiment and sample collections in the course of malting. Sample J0 represents the initial control. Samples P1–P4 correspond to PEF-treated barley, while samples N1–N4 represent non-treated barley, both undergoing successive malting stages, including pre-soaking, steeping, and germination. A 0.05 M phosphate buffer was used during the pre-soaking step.

For the malting, an automatic micromalting system was used, which includes three separate chambers for steeping, germination, and kilning. One kilogram of control barley and one kilogram of PEF-treated barley were processed for malting. The Pilsen-type malt process was as follows: steeping for 48 h at 15 °C, germination for 72 h at 15 °C with 95–98% humidity, and kilning for 24 h with a drying air temperature gradient ranging from 45 °C to 82 °C. The schematic of the samples collected during malting is illustrated in Figure 1.

### 2.4. Fusarium Species Analysis

DNA isolation and subsequent real-time PCR assays for *Fusarium* species analysis were conducted as described by Prusova et al. [35] and Ovesna et al. [36]. Briefly, DNA was extracted from three biological replicates per sample, and RT-PCR assays were performed in triplicate using TaqMan^®^ Universal PCR MasterMix (ThermoFisher Scientific, Waltham, MA, USA), species-specific primers and probes (Table S1). Amplification was carried out on a QuantStudio^TM^ 6 cycler (ThermoFisher Scientific, Waltham, MA, USA) under standard thermal cycling conditions. *Fusarium* DNA quantification was based on a standard dilution series (0.9–1000 ng), with Ct values interpolated from regression equations. Fungal DNA content was expressed as a ng of DNA per mg of dry matter (Table S2).

### 2.5. Sampling and RNA Isolation

RNA samples were collected from all stages of both treatment groups. All barley samples were collected in 20 g quantities, snap-frozen immediately in liquid nitrogen, and stored at −180 °C until further analysis. RNA was extracted from the frozen material using the TRIzol reagent (Thermo Fisher Scientific, Waltham, MA, USA) and purified with an RNeasy column (QIAGEN, Hilden, Germany) in the presence of DNase (QIAGEN, Hilden, Germany). The quality of RNA was assessed using agarose gel electrophoresis and the 4200 TapeStation System (Agilent Technologies, Santa Clara, CA, USA). Each sample was represented by three independent replicates.

### 2.6. New Generation Sequencing Analysis

To ensure the integrity and quality of the RNA samples for subsequent New Generation Sequencing (NGS) analysis, a secondary check was performed at a company providing NGS analysis (Seqme, Prague, Czech Republic). This step confirmed that the samples met the required standards. Regarding the NGS analysis, 3′ mRNA sequencing was chosen. Next, sequencing libraries were prepared for each biological replicate of every sample.

The Lexogen QuantSeq 3′ mRNA-Seq Library Prep Kit FWD (Lexogen, Vienna, Austria) for Illumina, along with Lexogen Unique Molecular Identifiers and the Lexogen UDI 12 nt Unique Dual Indexing Add-on Kit, were used to prepare RNA-Seq libraries by targeting the 3′ ends of polyadenylated RNA. The process began with the selective capture of mRNA using oligo priming, which simplified transcriptome sequencing and provided a more accurate representation of gene expression levels. UMIs were incorporated into each cDNA molecule to enable precise error correction and reduce amplification bias, enhancing the quantification of low-abundance transcripts. The dual indexing kit provided unique combinations of indices for each library, allowing for the multiplexing of samples in a single sequencing run, improving the accuracy of sample identification, and minimizing cross-contamination. Together, these reagents facilitated the generation of high-quality RNA-Seq data with reduced technical variability and reliable expression profiling. Following quantification, individual libraries were normalized to compensate for differences in RNA concentration and pooled for further analysis, ensuring consistent sequencing depth across samples.

The library quality control was performed using the Agilent Bioanalyzer 2100 High Sensitivity DNA Kit (Agilent Technologies, Santa Clara, CA, USA), Invitrogen Collibri Library Quantification Kit (Thermo Fisher Scientific, Waltham, MA, USA), and Qubit 1X dsDNA High-Sensitivity Assay Kit (Thermo Fisher Scientific, Waltham, MA, USA) to ensure the integrity and accuracy of the RNA-Seq libraries. The Agilent Bioanalyzer 2100 High Sensitivity DNA Kit was employed to evaluate the size distribution and concentration of the DNA fragments, allowing for the identification of any library preparation issues such as adapter dimers or incomplete reactions. The Invitrogen Collibri Library Quantification Kit was used to precisely quantify the libraries, ensuring optimal cluster density and accurate loading for sequencing. The Qubit 1X dsDNA High-Sensitivity Assay Kit provided additional quantification of the double-stranded DNA, offering a highly sensitive and specific measurement of DNA concentration. Together, these reagents ensured that the RNA-Seq libraries were of high quality, accurately quantified, and ready for successful sequencing.

The sequencing itself was performed using the Illumina Novaseq platform (Illumina, San Diego, CA, USA) with the following parameters: single-end sequencing, 50 base pairs per read, and a sequencing depth of 100 million reads

### 2.7. Data Treatment

#### 2.7.1. Data Preprocessing

During the sequencing process, raw fastq files were generated for each sample. These files underwent data preprocessing to ensure quality and remove unwanted sequences. Initial quality assessment was conducted using FastQC [37] (v. 0.12.1) and multiQC [38] (v. 1.25) programs. Trim Galore was then employed to eliminate low-quality reads and adapter sequences. The resulting data were again evaluated using FastQC and multiQC.

#### 2.7.2. Mapping to Reference

After preprocessing, the reads were mapped to barley genome reference (MorexV3_pseudomolecules_assembly; GCA_904849725.1) using Hisat2 [39].

#### 2.7.3. Differential Expression Analysis

To analyze differential expression, the mapped reads were utilized. The count of mapped reads was obtained using featureCounts [40] (v2.0.8) and stored in a count matrix. Differential expressed genes (DEGs) were determined using DESeq2 [41] (v 1.46.0). Transcripts with a log2-fold change greater than 1 or less than −1 and a *p*-value less than 0.05 were considered significantly differentially expressed. Pairwise comparisons were made between all samples from the PEF (P1–P4) and control groups (N1–N4) against input barley (J0) to determine the presence of differentially expressed genes. Furthermore, comparisons were performed vertically (in the course of malting) and horizontally (between treatments) to assess the number of DEGs.

#### 2.7.4. Gene Enrichment Analysis

The sets of DEGs were subjected to Gene Ontology (GO) enrichment analysis. GO identifiers for individual DEGs were obtained from the NCBI database (https://www.ncbi.nlm.nih.gov/datasets/genome/GCF_904849725.1/, accessed on 3 January 2025). The sets of GO identifiers for the groups of DEGs were analyzed using the ClusterProfiler package [42]. Enriched categories with a *p*-value less than 0.05 were considered over-represented, indicating significant biological processes or pathways associated with the groups of DEGs.

## 3. Results

Based on the specified thresholds, a total of 12,109 DEGs were identified in *Hordeum vulgare* across various pairwise comparisons of interest. 

### 3.1. Transcripton Changes in the Course of Malting

**Figure 2:**
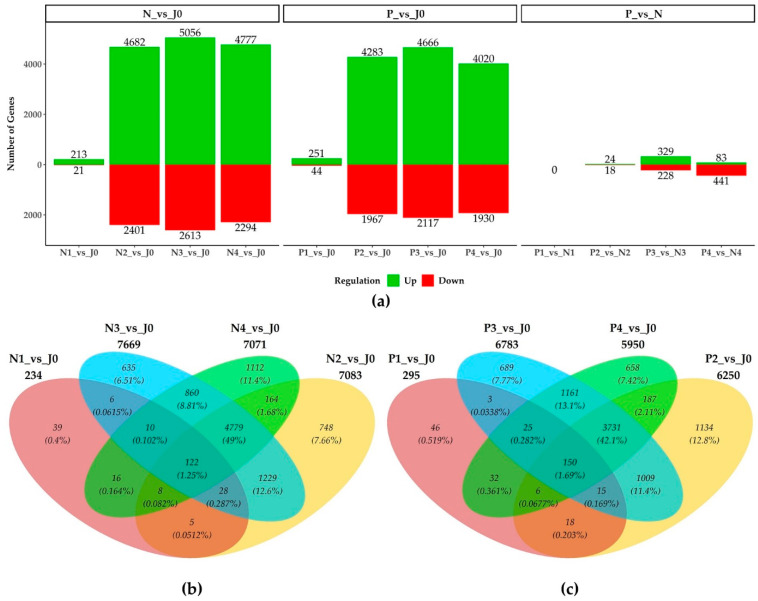
Number of DEGs identified in pairwise comparisons of analyzed barley samples. Transcripts from all examined stages were compared separately. (**a**) The number of up-regulated and down-regulated genes is displayed, along with (**b**,**c**) Venn diagrams showing the numbers of DEGs specific to or shared between (**b**) non-treated and (**c**) PEF-treated samples, compared with the J0 sample.

A comparison of PEF-treated and non-treated samples with input barley (J0) revealed that the third stage of malting (germination after 24 h) exhibited the highest number of DEGs (6783) (Figure 2c), followed by the second stage (steeping; 6250) and the fourth stage (end of germination; 5950). Approximately 1.7% of all DEGs were shared across all four samples, while around 44% were shared among the P2, P3, and P4 samples.

For non-treated samples, the order of malting phases based on the number of differentially expressed genes followed a similar pattern when compared with J0 (Figure 2b). The highest number of DEGs was observed in the third stage of malting (7669), followed by the second stage (7083) and the fourth stage (7071). Approximately 1.25% of all DEGs were shared across all four samples, and more than 50% were shared among the N2, N3, and N4 samples.

It should be noted that, when PEF-treated samples were compared with J0, significantly fewer genes were differentially expressed compared to non-treated samples, except during the first phase (pre-soaking) of malting. This suggests that the effect of PEF becomes more pronounced in the later phases of the malting process.

**Figure 3:**
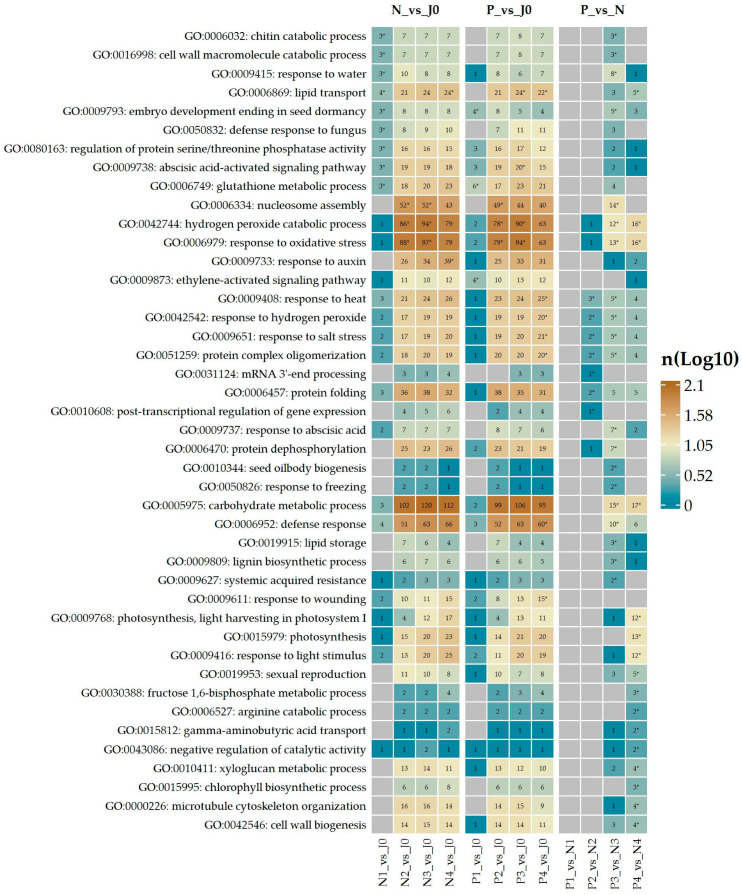
GO terms enriched in sets of DEGs resulting from various pairwise comparisons of the samples of interest. The number of genes involved in each category is displayed on a log10 scale. Asterisks highlight the GO terms that are significantly enriched in each respective comparison.

The GO enrichment analysis reveals dynamic changes in the number of involved genes across different comparisons (Figure 3), reflecting the shifting biological processes as the barley progresses through the malting stages and responds to *Fusarium* infection. Several key processes, such as response to oxidative stress and hydrogen peroxide catabolic process, show an increasing number of involved genes in comparisons like P_vs_J0 and N_vs_J0, indicating a growing activation of oxidative defense mechanisms as the stress intensifies. Similarly, the defense response to fungus and abscisic acid-activated signaling pathway also exhibit an increasing number of genes involved, particularly in P_vs_N and P_vs_J0, suggesting an escalating pathogen defense and stress signaling as malting progresses.

On the other hand, processes like photosynthesis and light harvesting in photosystem I show a decreasing number of involved genes, particularly in N_vs_J0 and P_vs_J0, reflecting the suppression of photosynthetic activity during malting and pathogen stress. Additionally, nucleosome assembly, which is enriched in N_vs_J0 and P_vs_J0, shows a moderate increase in the number of involved genes, indicating chromatin-related changes as a response to stress.

Overall, the results highlight a trend where stress-related processes such as oxidative stress, defense mechanisms, and hormonal signaling increasingly involve more genes, while processes related to photosynthesis and light harvesting experience a reduction in gene involvement, reflecting a shift in the plant’s priorities towards stress adaptation and survival.

### 3.2. Transcription Changes Between Treatmens

When comparing PEF-treated versus non-treated samples, the third stage of malting exhibited the highest number of DEGs, with 329 genes up-regulated and 228 down-regulated (Figure 2a). These trends suggest that the malting process and PEF treatment influence gene expression in a stage-specific manner, with the most significant changes occurring during the later stages of malting.

#### 3.2.1. Third Stage of Malting

**Figure 4:**
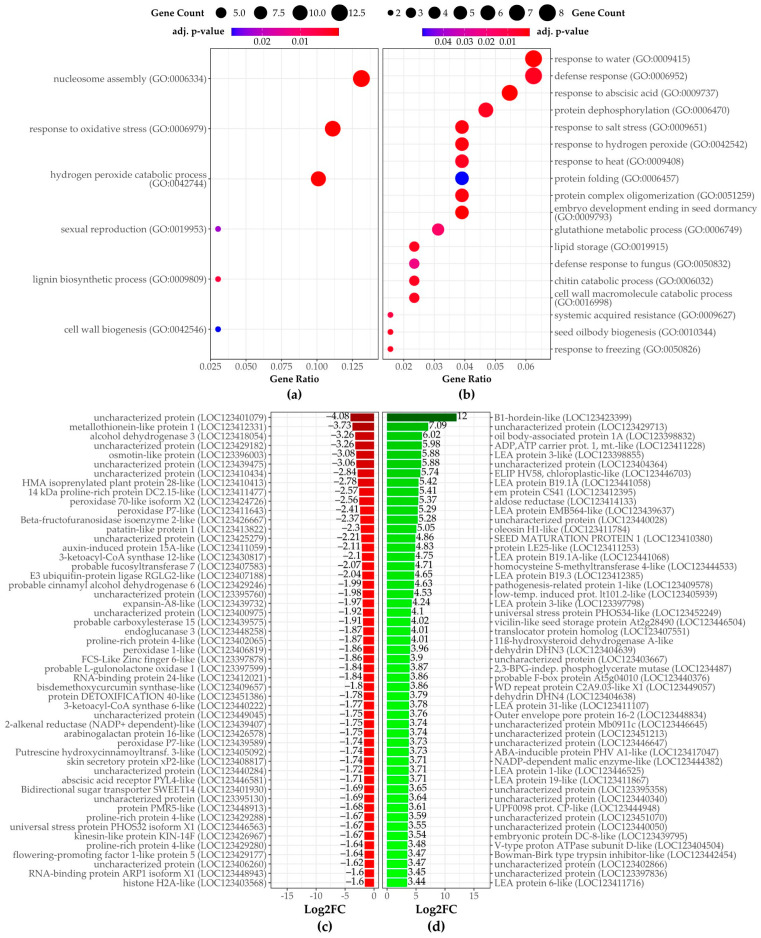
Over-represented GO terms for biological processes are shown for genes that are (**a**) down-regulated and (**b**) up-regulated in the P3 vs. N3 comparison. Dot size is proportional to the number of genes associated with each GO term, while dot color indicates the enrichment significance based on the adjusted *p*-value. The most significantly (**c**) down- and (**d**) up-regulated genes between P3 and N3 samples are also displayed, including the magnitude of transcriptional change and its significance. ‘Description’ refers to the functional annotation of the respective protein encoded by each DEG.

Enriched GO terms identified for the group of down-regulated DEGs (Figure 4a) include nucleosome assembly, represented by various histone family genes (Figure S1), and response to oxidative stress (Figure S2) or hydrogen peroxide catabolic process (Figure S3), primarily involving several peroxidases. Another enriched group involves genes associated with lignin biosynthetic processes (Figure S4), such as cinnamyl alcohol dehydrogenases, and cell wall biogenesis (Figure S4), which includes xyloglucan endotransglycosylase/hydrolase and fucosyltransferases. Among the most strongly down-regulated genes are those encoding enzymes involved in biosynthesis of very long chain fatty acids, proteins related to pathogenesis and osmotic stress, as well as proteins involved in detoxification and cellular antioxidative defense (Figure 4c; Table S3). Other significantly down-regulated genes include those related to the lipid metabolic process, cell wall organization, and genes induced by auxin stimulus (Figure S5).

**Figure 5:**
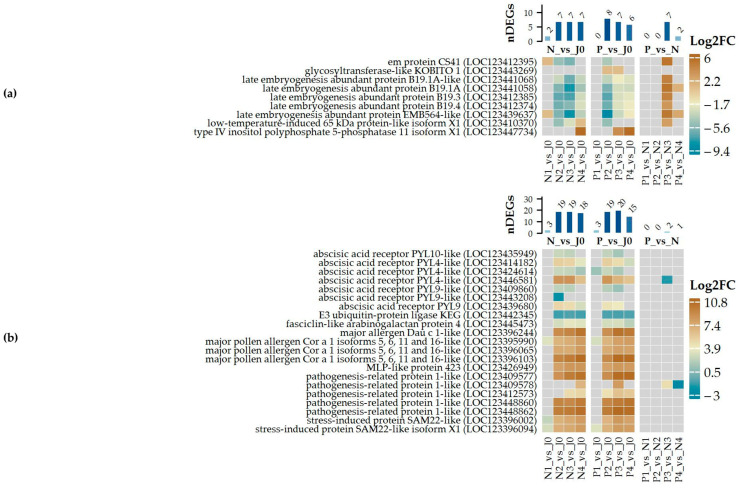
Differentially expressed genes (DEGs) associated with (**a**) Response to ABA (GO_0009737) and (**b**) ABA activated signaling (GO_0009738). Log2-transformed expression changes are displayed for selected pairwise comparisons, along with the number of DEGs identified in each specific category for the respective comparison. The magnitude of expression change is represented by the color scale shown on the right side of the plot.

**Figure 6:**
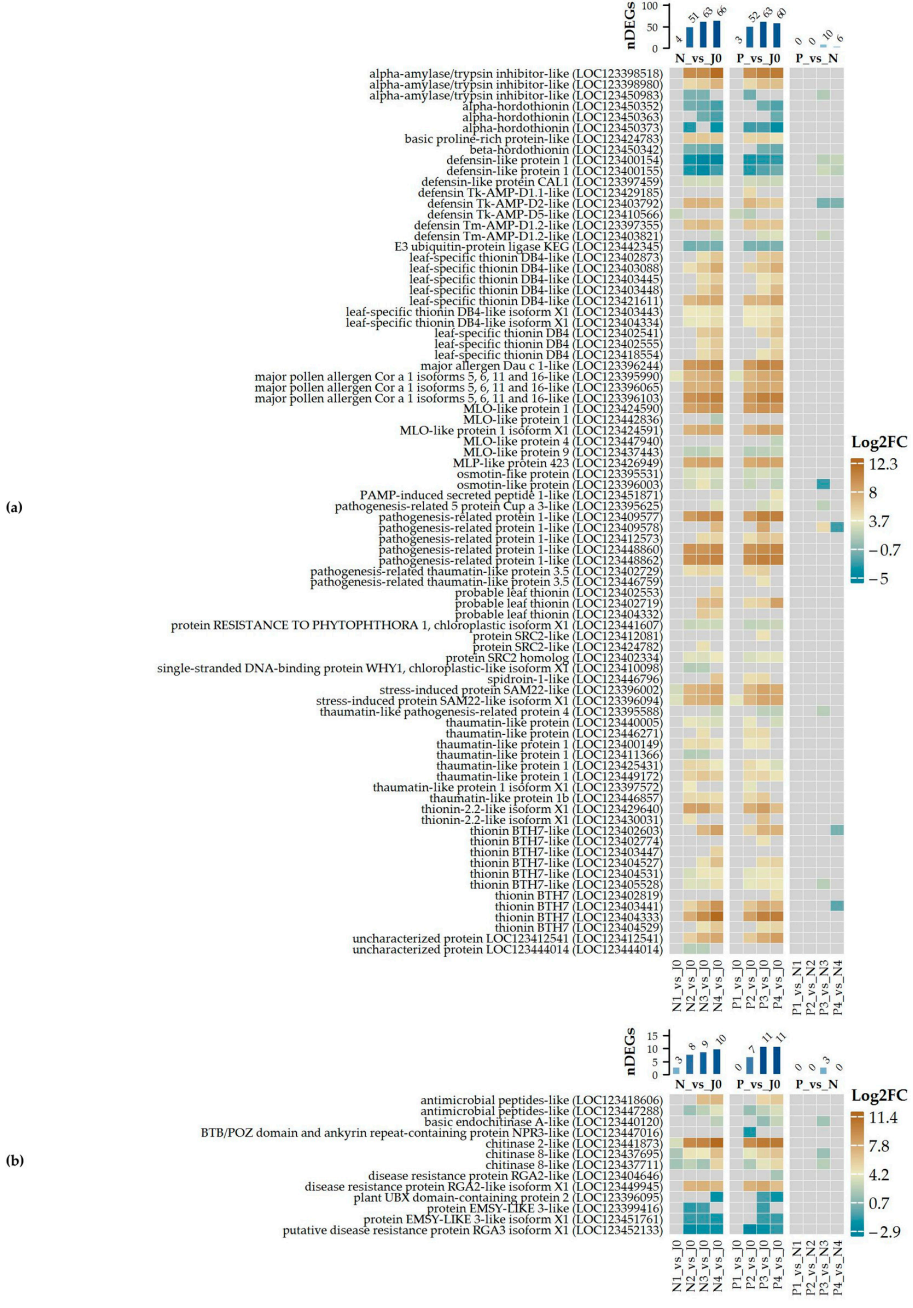
Differentially expressed genes (DEGs) associated with (**a**) Defense (GO_0006952) and (**b**) Defense response to fungus (GO_0050832). Log2-transformed expression changes are displayed for selected pairwise comparisons, along with the number of DEGs identified in each specific category for the respective comparison. The magnitude of expression change is represented by the color scale shown on the right side of the plot.

For up-regulated transcripts (Figure 4b), response to water and response to abscisic acid (Figure 5) are strongly enriched. The former is represented by several cold-shock proteins and members of the dehydrin protein family, while the latter includes late embryogenesis abundant proteins such as LEA B191A, B19.1A-like, B19.3, B19.4, EMB564-like, or HVA1 (Figure 4d; Table S3). Many of these genes are among the most profoundly up-regulated in the P3_vs_N3 comparison. Additionally, the enriched defense response category includes genes coding for defensins, alpha-amylase/trypsin inhibitors, pathogenesis-related proteins, thaumatin-like proteins, and thionins such as BTH7 (Figure 6). Defense-related terms also include defense response to fungi, with several chitinases involved in cell wall macromolecule catabolic processes (Figure 6). Beyond these processes, other enriched terms include response to heat (Figure S6) and response to hydrogen peroxide (Figure S3), which account for several small heat-shock proteins, as well as terms related to lipid storage and seed oil body biogenesis, represented by oleosins. Finally, systemic-acquired resistance includes two non-specific lipid transfer proteins.

#### 3.2.2. Fourth Stage of Malting

**Figure 7:**
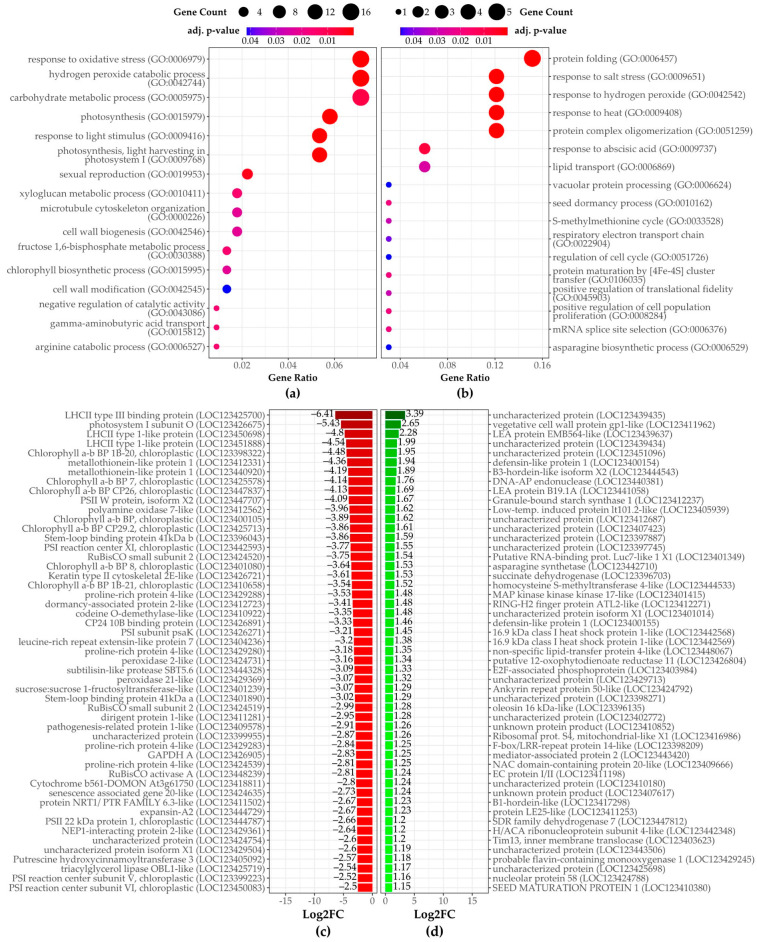
Over-represented GO terms for biological processes are shown for genes that are (**a**) down-regulated and (**b**) up-regulated in the P4 vs. N4 comparison. Dot size is proportional to the number of genes associated with each GO term, while dot color indicates the enrichment significance based on the adjusted *p*-value. The most significantly (**c**) down- and (**d**) up-regulated genes between P4 and N4 samples are also displayed, including the magnitude of transcriptional change and its significance. ‘Description’ refers to the functional annotation of the respective protein encoded by each DEG.

**Figure 8:**
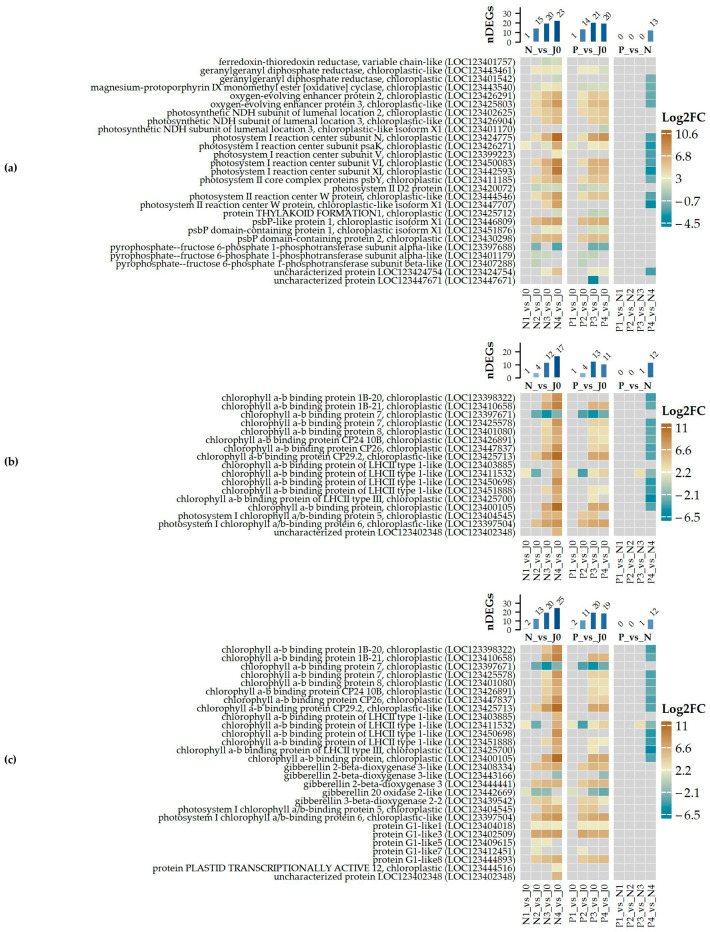
Differentially expressed genes (DEGs) associated with (**a**) Photosynthesis (GO_0015979), (**b**) Photosynthesis light harvesting in photosystem I (GO_0009768), and (**c**) Response to light stimulus (GO_0009416). Log2-transformed expression changes are displayed for selected pairwise comparisons, along with the number of DEGs identified in each specific category for the respective comparison. The magnitude of expression change is represented by the color scale shown on the right side of the plot.

In the fourth stage of malting, down-regulated genes were highly enriched in several biological processes (Figure 7a), many of which overlap with those identified in the third stage of malting (Figure 3 and Figure 4) such as “Hydrogen peroxide catabolic process” (Figure S3) or “response to oxidative stress” (Figure S2). However, a notable addition in the fourth stage is the enrichment of terms related to photosynthesis (Figure 8), with many down-regulated chloroplast-associated proteins such as chlorophyll a-b binding protein family members.

Additionally, ‘fructose 1,6-bisphosphate metabolic process’ and ‘photosynthesis, light reaction’ have emerged, indicating reduced activity in the energy metabolism processes that are fundamental to plant function. This reduction in photosynthetic machinery was not seen in the third stage of malting and highlights the progressive response of the barley sample to the treatment as malting continues or it is the response to *Fusarium* infection.

Down-regulated transcripts also continued to show a decrease in proteins linked to cell wall biogenesis like xyloglucan endotransglycosylase, observed in P3 vs. N3. Genes related to lipid metabolism and detoxification, such as triacylglycerol lipase 1 and metallothionein-like proteins, are among the most strongly down-regulated genes (Figure 7c; Table S4), pointing towards suppressed lipid metabolism and stress-related defense mechanisms in the late malting stages.

The up-regulated DEGs in P4 vs. N4 reflect a continued dominance of stress-response-related processes (Figure 7b), with significant enrichment for “response to salt stress” (Figure S6), “response to heat” (Figure S6), and “response to abscisic acid” (Figure 5), just as in the third stage (Figure 3 and Figure 4). These processes are primarily driven by the up-regulation of small heat shock proteins and late embryogenesis abundant proteins (Figure 7d, Table S4). Protein folding, involving heat shock proteins, emerges as a key process, suggesting an increased need to manage protein homeostasis under stress. Response to hydrogen peroxide catabolic processes, which were also prominent in the third stage, remain critical in the fourth stage, involving the up-regulation of small heat shock proteins. Its increased expression likely aids in maintaining protein stability under stress. Other up-regulated DEGs included *asparagine synthetase* and *homocysteine S-methyltransferase*, highlighting the activation of pathways involved in nitrogen metabolism and stress-related defense during the late stages of malting. Continuing up-regulation of genes such as late embryogenesis abundant proteins, defensins, and additional heat shock proteins, further indicates a stronger activation or lesser inhibition of stress tolerance pathways in response to malting and *Fusarium* infection.

## 4. Discussion

The results of this study provide important insights into the transcriptional changes occurring in *Hordeum vulgare* during the malting process, particularly in response to PEF treatment and *Fusarium* infection. The identification of 12,109 differentially expressed genes across various pairwise comparisons underscores the extensive impact of the malting process on gene expression. The most significant changes were observed in the third stage (germination for 24 h) of malting, with N3_vs_J0 and P3_vs_J0 showing the highest number of DEGs, highlighting this stage as a critical period for transcriptional activity. This observation aligns with previous findings that indicated the malting process is characterized by dynamic gene expression patterns, particularly during germination and early growth phases, which are crucial for the development of malting quality traits in barley [43,44].

One key finding is that PEF-treated samples consistently showed fewer DEGs compared to non-treated samples, except in the first stage of malting. This suggests that PEF treatment may exert a moderating effect on gene expression, potentially reducing stress during the malting process. The PEF treatment’s role in modulating gene expression has been documented in other studies, where it was shown to enhance the stability of cellular responses and reduce oxidative stress [45,46]. In the P3_vs_N3 comparison, the 557 DEGs included 329 up-regulated and 228 down-regulated genes, indicating a substantial, stage-specific response. The up-regulated genes were enriched in stress response processes such as the response to water and abscisic acid. The presence of dehydrin proteins and late embryogenesis abundant proteins points to mechanisms aimed at enhancing cellular protection under osmotic and dehydration stresses typical of the malting environment, which is consistent with findings that highlight the importance of these proteins in plant stress responses [47,48]. These findings align with observations of signaling pathways linked to drought stress, where LEA proteins and dehydrins are expressed and closely associated with ABA [49]. While ABA can suppress plant defense responses against biotic stresses, such as fungal pathogens, it also plays a key role in mitigating abiotic stresses [50]. Notably, drought response pathways have previously been shown to contribute to resistance against *Fusarium* infection [51,52]. Despite assumptions that PEF treatments may inhibit ABA pathways, recent evidence suggests that PEF positively influences the resistance of germinating barley to salinity stress—an abiotic challenge with effects similar to drought [53]. Pulsed electric field (PEF) is well documented for inducing temporary membrane permeabilization through electroporation, leading to increased ion flux and water uptake in plant cells. This mild physical stress acts as a priming stimulus, triggering cellular responses that enhance the ability of plant seeds to adapt to subsequent abiotic stress conditions [54]. Such findings align with our observations, suggesting that PEF treatment can serve as an initial signal to activate adaptive mechanisms during malting, thereby improving the overall stress tolerance of barley.

The down-regulation of genes associated with cell wall biogenesis and lipid metabolism, including expansin-A8-like proteins and triacylglycerol lipase 1, reflects a potential shift in metabolic priorities. This reallocation may favor defensive processes over growth, aligning with a plant’s strategic adaptation to stress. The involvement of oxidative stress responses, such as those mediated by peroxidases and other antioxidative enzymes, further supports the notion that managing reactive oxygen species is critical during these stages [55,56]. The continued expression of heat shock proteins and metallothionein-like proteins underlines barley’s effort to maintain protein stability and mitigate damage from both biotic and abiotic stresses, reinforcing the protective roles these proteins play in stress resilience [48,57].

The fourth stage of malting presented a continuation of stress response trends, with additional down-regulation of photosynthesis-related genes, such as chlorophyll a-b binding proteins. This suggests a progressive adaptation strategy where energy-intensive processes like photosynthesis are reduced in favor of stress tolerance. The emergence of GO terms related to ‘photosynthesis’ and ‘light harvesting’ among down-regulated DEGs highlights a shift in metabolic focus as the plant allocates resources to counteract prolonged stress, a phenomenon that has been documented in other studies examining plant responses to environmental stressors [58,59]. PEF treatment’s influence on the transcriptional landscape becomes evident when considering the smaller number of DEGs in PEF-treated samples during later malting stages. This indicates that PEF may modulate gene expression to stabilize stress responses, potentially reducing the expression of genes related to oxidative stress pathways while maintaining necessary stress adaptations.

The biological implications of these findings are significant. The enriched GO terms related to defense responses, such as ‘defense response to fungus’ and ‘abscisic acid-activated signaling pathway’ underscore barley’s integrated response to *Fusarium* infection during malting. The data suggest that the malting process, combined with external stressors, triggers a comprehensive stress response involving both ROS management and hormonal signaling pathways. The consistent up-regulation of heat shock proteins and LEA proteins emphasizes their importance in managing protein folding and stability under stress, aligning with known protective roles in plant resilience [47,48,57]. These findings have practical implications for barley breeding and malting process optimization. Understanding the gene expression changes can inform breeding programs aimed at enhancing stress resilience. For instance, leveraging genetic markers associated with robust stress responses could guide the development of barley varieties better suited for challenging malting conditions [44,60].

## 5. Conclusions

This study demonstrates the complexity of transcriptional responses in *Hordeum vulgare* during the malting process, particularly under the influence of pulsed electric field (PEF) treatment and *Fusarium* infection. Across various malting stages, extensive differential gene expression revealed significant shifts in metabolic and defense-related pathways, with the third stage showing the highest transcriptional activity, underscoring its critical role in stress adaptation during malting.

Notably, PEF treatment exhibited a stabilizing effect on gene expression, as reflected by a reduced number of differentially expressed genes, particularly in the later malting stages. This effect may be attributed to temporary membrane permeabilization, which serves as a mild ‘priming effect’. By acting as an initial stress stimulus, PEF likely triggers adaptive cellular responses that enhance resilience to subsequent biotic and abiotic stressors, thereby contributing to improved stress management during malting.

These findings highlight the potential of PEF treatment to improve malt quality while mitigating stress impacts, providing a foundation for optimizing malting techniques. Furthermore, the identified stress-responsive pathways offer valuable targets for barley breeding programs aimed at enhancing resilience. Future research should focus on validating the molecular mechanisms underlying this response and expanding these findings to diverse barley varieties and *Fusarium* strains to broaden the applicability of PEF treatment in malting practices.

## Data Availability

Further information is available from the corresponding authors upon reasonable request.

## Supplementary Materials

The following supporting information can be downloaded at https://www.mdpi.com/article/10.3390/plants14050668/s1, Table S1: Real-time primers and probes used for the quantification of *Fusarium* species DNA [61,62,63,64]. Table S2: Fungal DNA content expressed in ng of DNA per mg of Dry matter. Table S3: The most significantly (c) down- and (d) up-regulated genes between P3 and N3 samples. Table S4: The most significantly (c) down- and (d) up-regulated genes between P4 and N4 samples. Figure S1: Differentially expressed genes (DEGs) associated with the response to nucleosome assembly (GO_0006334). Figure S2: Differentially expressed genes (DEGs) associated with the response to oxidative stress (GO_0006979). Figure S3: Differentially expressed genes (DEGs) associated with the response to hydrogen peroxide catabolic process (GO_0042744). Figure S4: Differentially expressed genes (DEGs) associated with (a) Cell wall biogenesis (GO_0042546), (b) Cell wall macromolecule catabolic process (GO_0016998) and (c) Lignin biosynthesis (GO_0009809). Figure S5: Differentially expressed genes (DEGs) associated with the response to auxin (GO_0009733). Figure S6: Differentially expressed genes (DEGs) associated with (a) Response to heat (GO_0009408), (b) Response to water (GO_0009415) and (c) Response to freezing (GO_0050826).
