# Supplementary material for: Pulsed Electric Field Treatment Modulates Gene Expression and Stress Responses in Fusarium-Infected Malting Barley
Source: Plants (Basel). 2025 Feb 21;14(5):668. doi: 10.3390/plants14050668 (PMC11901457; doi:10.3390/plants14050668)
Supplement: Supplementary file 1 [file plants-14-00668-s001.zip › Figure S1-6.pdf]

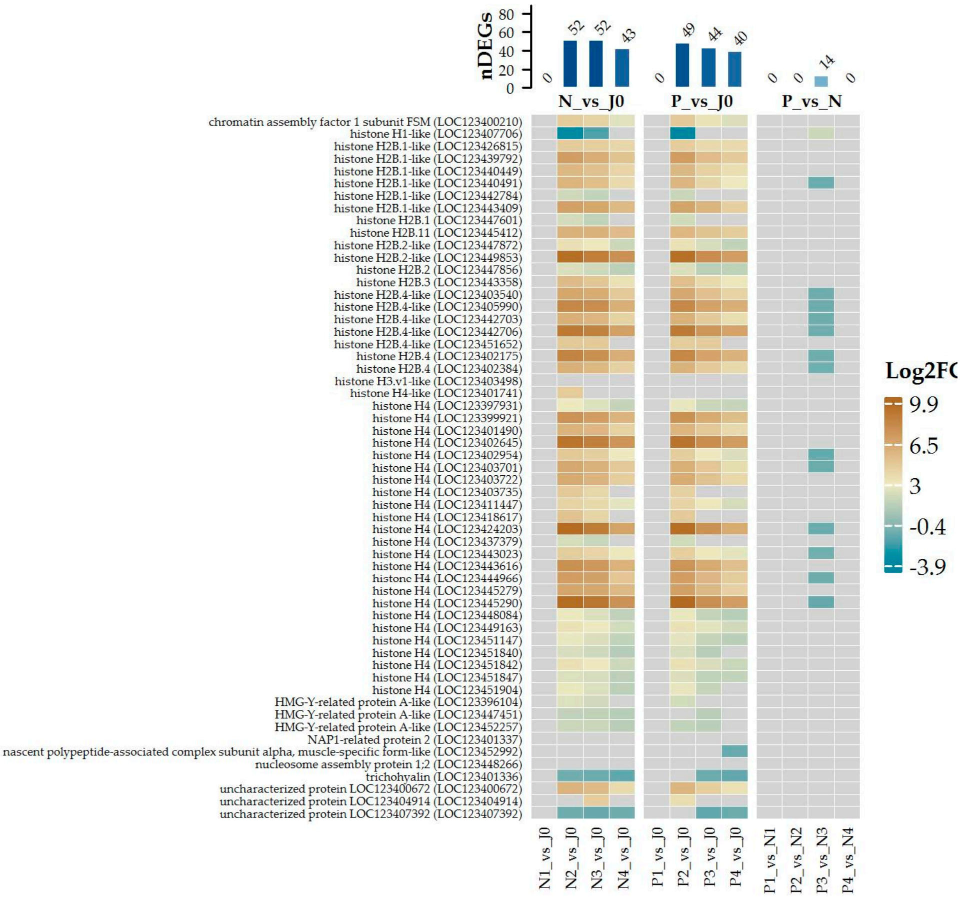

Fig. S1: Differentially expressed genes (DEGs) associated with the response to nucleosome assembly (GO_0006334). Log2-transformed expression changes are displayed for selected pairwise comparisons, along with the number of DEGs identified in each specific category for the respective comparison. The magnitude of expression change is represented by the color scale shown on the right side of the plot.

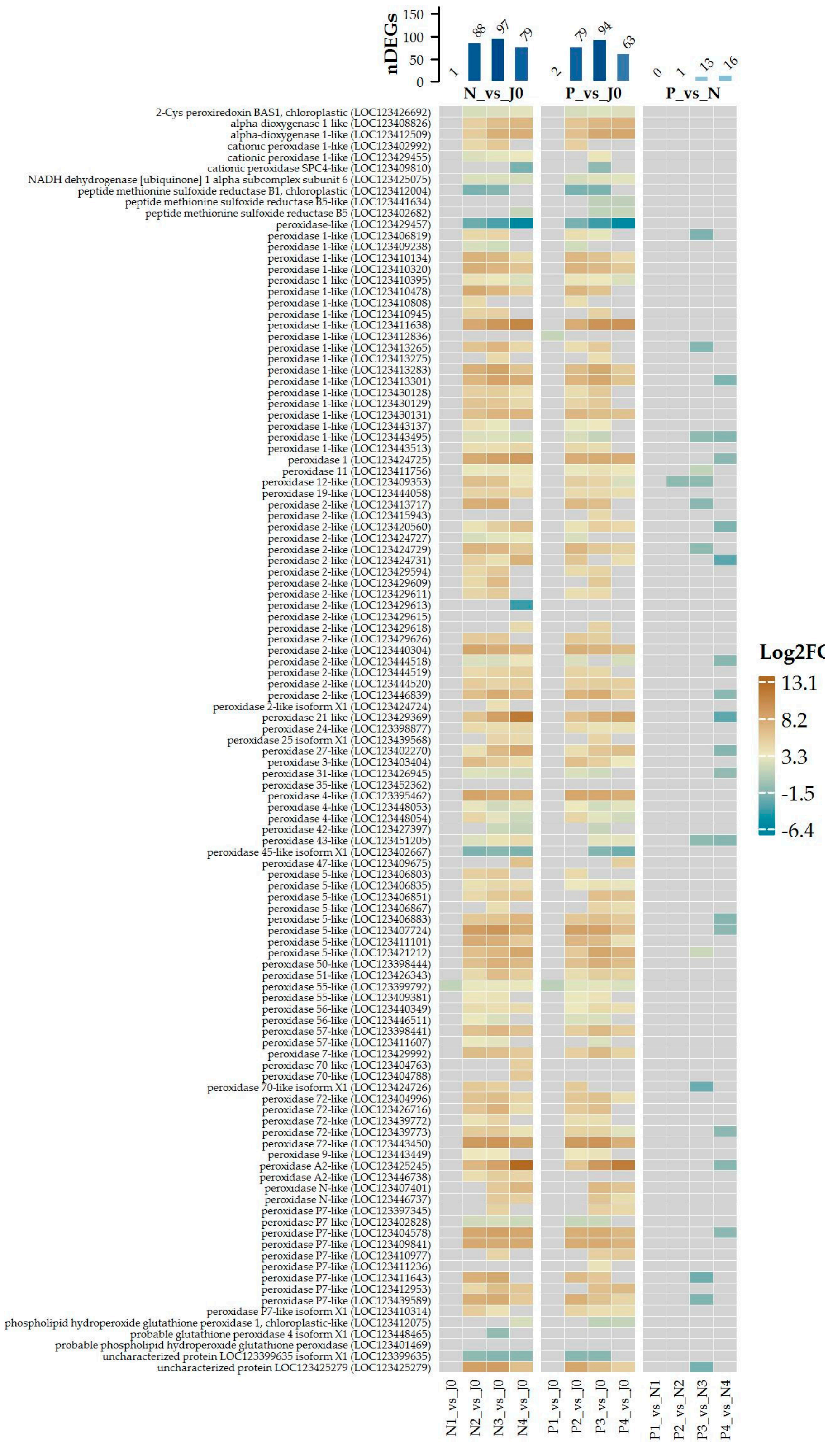

Fig. S2: Differentially expressed genes (DEGs) associated with the response to oxidative stress (GO_0006979). Log2-transformed expression changes are displayed for selected pairwise comparisons, along with the number of DEGs identified in each specific category for the respective comparison. The magnitude of expression change is represented by the color scale shown on the right side of the plot.

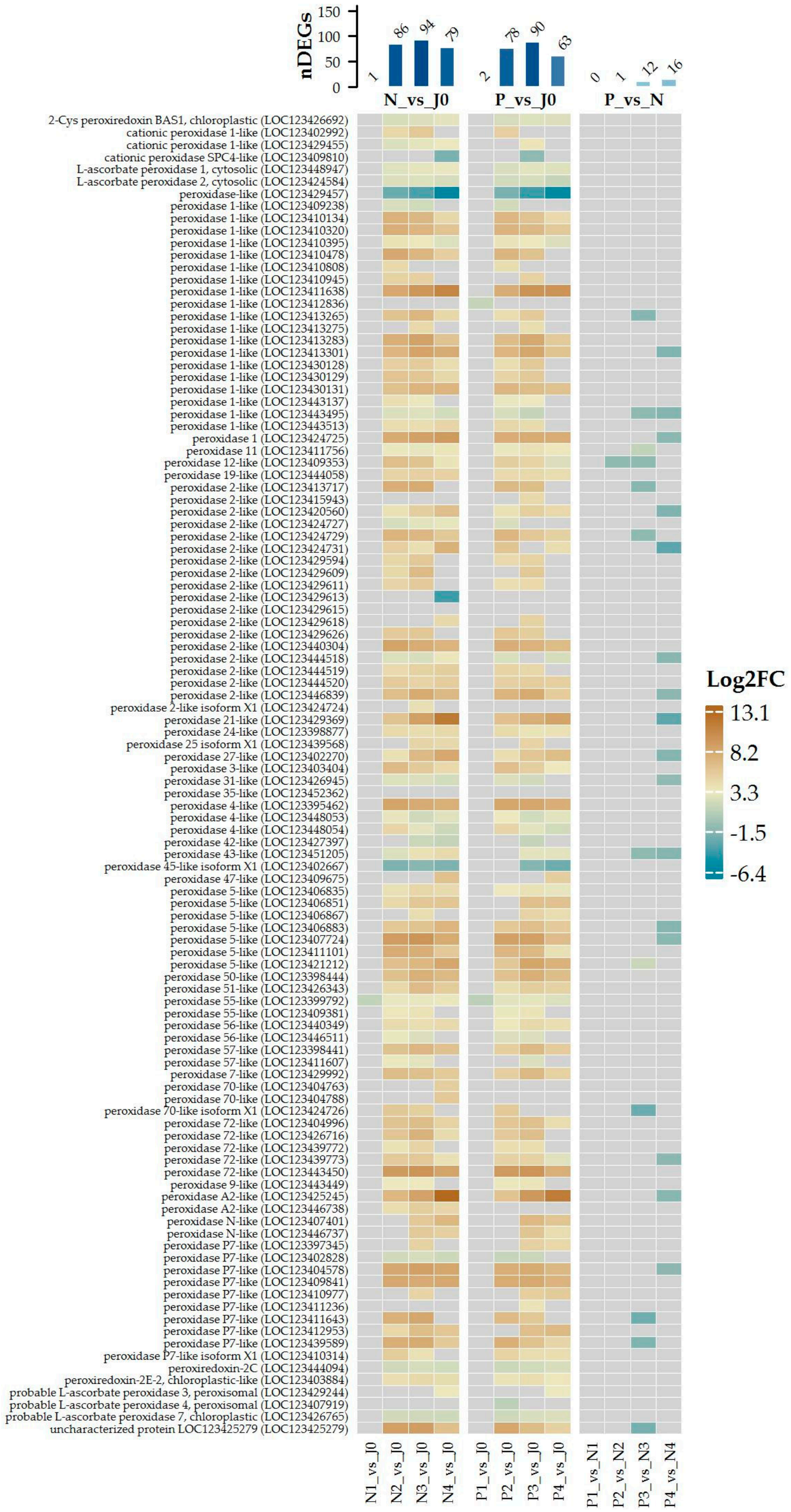

Fig. S3: Differentially expressed genes (DEGs) associated with the response to hydrogen peroxide catabolic process (GO_0042744). Log2-transformed expression changes are displayed for selected pairwise comparisons, along with the number of DEGs identified in each specific category for the respective comparison. The magnitude of expression change is represented by the color scale shown on the right side of the plot.

(a)

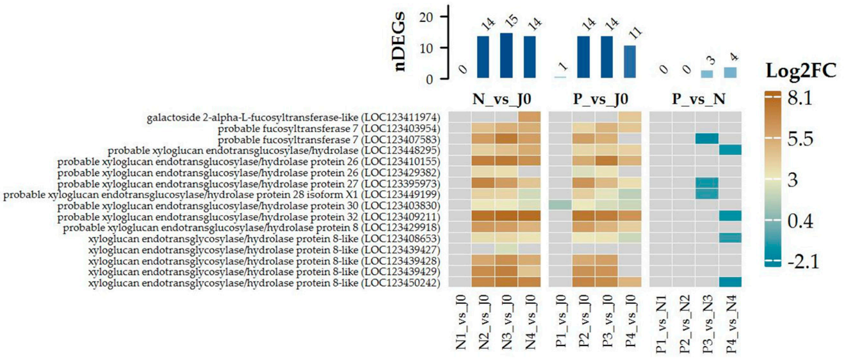

(b)

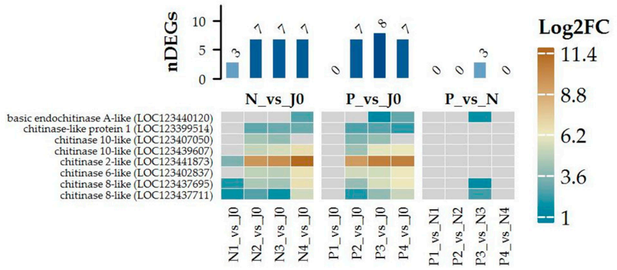

(c)

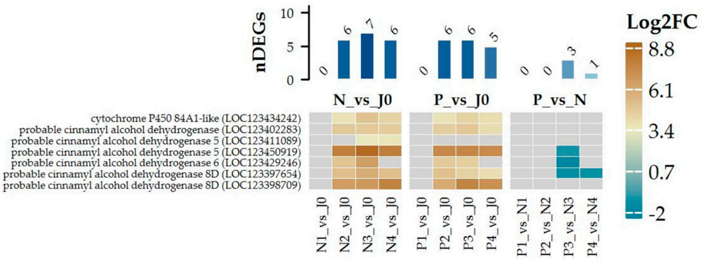

Fig. S4: Differentially expressed genes (DEGs) associated with a) Cell wall biogenesis (GO_0042546), b) Cell wall macromolecule catabolic process (GO_0016998) and c) Lignin biosynthesis (GO_0009809). Log2-transformed expression changes are displayed for selected pairwise comparisons, along with the number of DEGs identified in each specific category for the respective comparison. The magnitude of expression change is represented by the color scale shown on the right side of the plot.

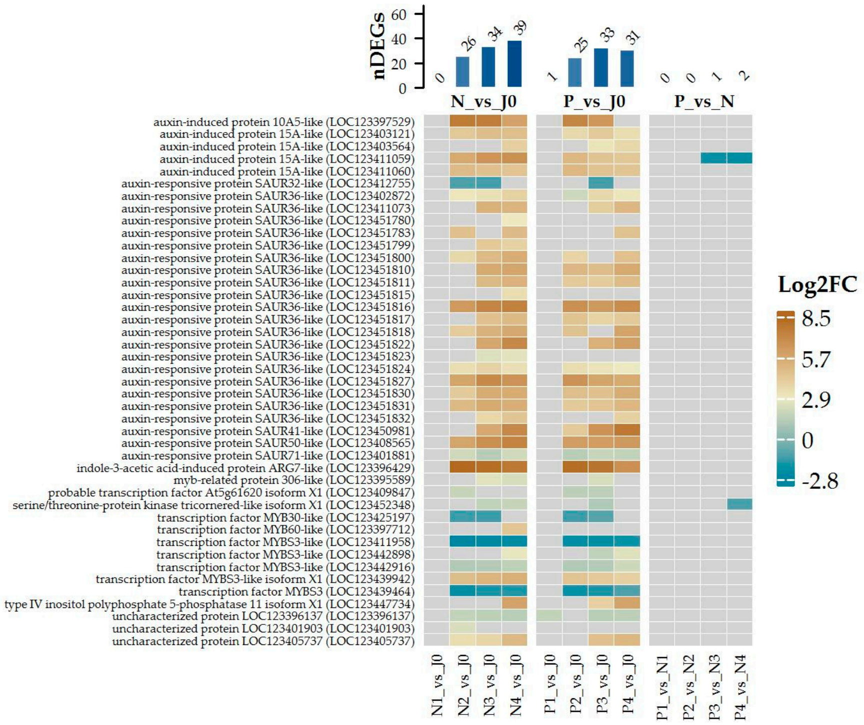

Fig. S5: Differentially expressed genes (DEGs) associated with the response to auxin (GO_0009733). Log2-transformed expression changes are displayed for selected pairwise comparisons, along with the number of DEGs identified in each specific category for the respective comparison. The magnitude of expression change is represented by the color scale shown on the right side of the plot.

(a)

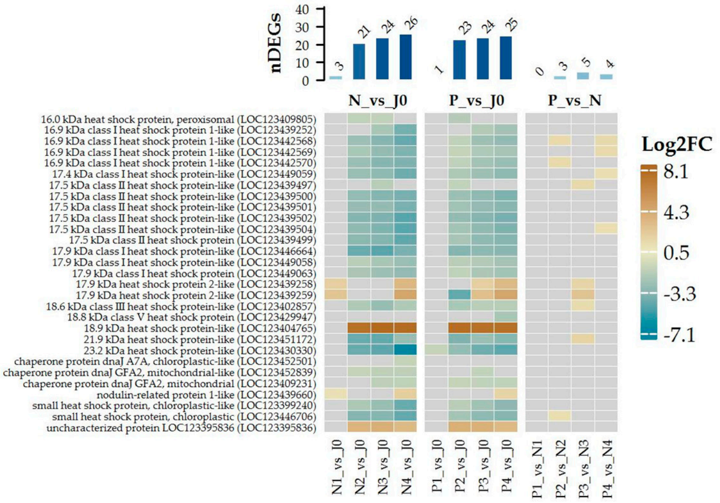

(b)

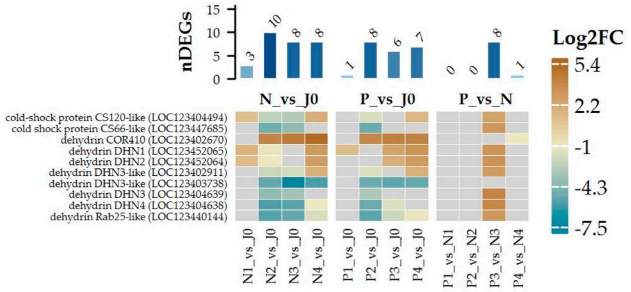

(c)

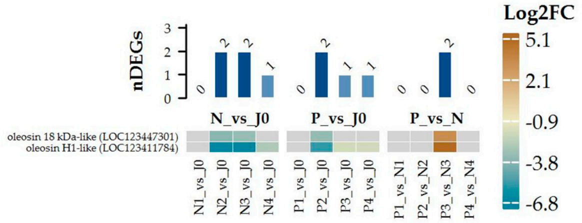

Fig. S6: Differentially expressed genes (DEGs) associated with a) Response to heat (GO_0009408), b) Response to water (GO_0009415) and c) Response to freezing (GO_0050826). Log2-transformed expression changes are displayed for selected pairwise comparisons, along with the number of DEGs identified in each specific category for the respective comparison. The magnitude of expression change is represented by the color scale shown on the right side of the plot.
