# Supplementary material for: Pulsed Electric Field Treatment Modulates Gene Expression and Stress Responses in Fusarium-Infected Malting Barley
Source: Plants (Basel). 2025 Feb 21;14(5):668. doi: 10.3390/plants14050668 (PMC11901457; doi:10.3390/plants14050668)
Supplement: Supplementary file 1 [file plants-14-00668-s001.zip › Table S1.pdf]

Table S1. Real-time primers and probes used for the quantification of *Fusarium* species DNA

Name	Type	Sequence (5′–3′) – fluorochrom/quencher	Reference*
**F. culmorum**	Forward	TTCACTAGATGCTCGCGGAG	[61]
	Reverse	GAGCCCTCCAGCAGGGAAG	
	Probe	AAAGAGATTGCAGATTTGATGTG – VIC/MGB	
**F. graminearum**	Forward	CTCCGGATATGTTGCTGCTCAA	[62]
	Reverse	CGAAGATATCTCAGGATCCCA	
	Probe	TGAGATAGTTCTTGAGGCAGCATT – ABY/QSY	
**F. sporotrichioides**	Forward	GGTTGGGTCGTCTTACTTAC	[63]
	Reverse	AATTCTGACTCTGCATAATGGG	
	Probe	TGTTGCTCTTCAGCATAGGTTT – ABY/QSY	
**F. poae**	Forward	GCTAGGCGTAGCGGCTTCG	[62]
	Reverse	TCTGTCGGACGTTTCACGA	
	Probe	ATTCTCCCATTCGACGATCCGAGGA – ABY/QSY	
**Fusarium species (ITS)**	Forward	AACTCCCAAACCCGTGAAAAA	[64]
	Reverse	TTAAGCGGCTGCGGCG	
	Probe	CGCTCGAAAGAGTCGAAATGAACAATAAC – VIC/QSY	

*
