# Supplementary material for: Pulsed Electric Field Treatment Modulates Gene Expression and Stress Responses in Fusarium-Infected Malting Barley
Source: Plants (Basel). 2025 Feb 21;14(5):668. doi: 10.3390/plants14050668 (PMC11901457; doi:10.3390/plants14050668)
Supplement: Supplementary file 1 [file plants-14-00668-s001.zip › Table S2.pdf]

Table S1. Fungal DNA content expressed in ng of DNA per mg of Dry matter.

		F. culmorum	*F. gramin.*	*F. poae*	*F. sporotr.*	ITS
**Iput barley**		0.056001	0.062938	0.081051	0.060166	0.256404
**PEF**	steeping	0.019866	0.002115	0.01727	0.002679	0.051096
	germination		0.039854	0.023378	0.050015	0.365042
	end of germination	0.052902	0.019446	0.022077	0.024013	0.157171
**Non-treated**	steeping	0.003565	0.000884	0.008021	0.004038	0.018218
	germination	0.031818	0.018315	0.025436	0.020898	0.126837
	end of germination	0.110064	0.034124	0.034746	0.051142	0.309446
